# Zebrafish Models in Toxicology and Disease Studies

**DOI:** 10.3390/ijms25168608

**Published:** 2024-08-07

**Authors:** Ida Ferrandino

**Affiliations:** Department of Biology, University of Naples Federico II, Via Cinthia 21, 80126 Naples, Italy; ida.ferrandino@unina.it

*Danio rerio* is a small tropical freshwater fish, also known as *Brachydanio rerio* and commonly referred to as zebrafish, described for the first time in 1822 by Francis Hamilton in the Ganges River but widespread throughout the entire Great Himalayan region of Southeast Asia [1]. Zebrafish is among the most employed animal models for research in various biological disciplines. This fish was introduced in 1981 as a model organism in genetic studies [2] by George Streisinger et al. The use of zebrafish in experimental studies has significantly increased over the years for several reasons, having this model many advantages in comparison to other models and thus becoming incredibly popular. The focus of the following Special Issue “Zebrafish Models in Toxicology and Disease Studies” is on the benefits of employing zebrafish to study the molecular and cellular mechanisms of the harmful action of compounds known but also emerging contaminants associated with different human diseases and drugs. Today, the zebrafish model is seeing increasing use in biomedical research, including in numerous studies on metabolic disorders and cancer [3]; however, it has also become the forefront of toxicology research [4,5,6]. Indeed, this fish is also used not only for the detection of pollutants in water samples and for examination of their effects on the environment in eco-toxicological studies but also to analyze related diseases and investigate mechanisms of action and drug development [7,8,9]. Morphologically the zebrafish is a small teleost fish with a body length of 2–5 cm and its common name is for the horizontal blue stripes observable along its body. The female has an enlarged belly respect to the male, generally more slender and small and with a slightly yellow cast ventrally [1]. Their natural life is more than 5 years, and the lines commonly used in the laboratories can be wild, mutant, or transgenic [1,8]. This animal model provides significant advantages over the use of mice and rats, making it an interesting translational model [9]. Zebrafish have a high fertility rate per week. The development of zebrafish embryos is external, rapid, and visually easy to observe. Their embryos and chorion are transparent and this facilitates observation of development from the first moments of fertilization through to free swimming. Although humans may appear to be extremely different compared to zebrafish, many of their organs are markedly similar. Moreover, the fully sequenced genome of zebrafish shows 84% homology with the genes associated with human disease, and 70% of human genes have been found in zebrafish [3,8,9,10]. From a practical point of view, the success of zebrafish is also due to their maintenance and breeding in animal facilities, with their use requiring much less space and low costs with respect to other vertebrates such as rats or mice. Thus, there are numerous aspects that validate the use of this animal model in many scientific fields covering both basic and applied research, including medicine, toxicology, and environmental sciences. However, it is extremely important to take into account the necessity to optimize laboratory conditions to ensure animal welfare and improve scientific research [11].

Five articles and one review are the contributions presented in the following Special Issue regarding the use of zebrafish as an organism model in toxicology and disease studies. The published papers are relative both to embryos and adults of zebrafish and covers topics many different denoting the great versatile use of this model.

The review by Hussen et al. focuses on the use of zebrafish to study the toxicity of electronic cigarettes on the development and cardiovascular system [a]. In recent years, the use of electronic cigarettes has become widespread in response to the negative health effects of traditional cigarettes linked to lung cancer. Electronic cigarettes administer nicotine without tobacco combustion endorsing their safety. However, the knowledge of their effective impact on human health is lacking. Hussen et al. have provided a review of the state of research in general on electronic cigarettes and the specific use of zebrafish as a model to study their toxicity and highlight their potential impact on public health, in particular on vulnerable populations, such as pregnant women and fetuses. Electronic cigarette toxicity was analyzed in zebrafish through the study of cellular and molecular mechanisms in zebrafish embryos and adults through monitoring of development and behavior, showing the potential cardiovascular risks associated with their use. The authors remarked that although there are differences between zebrafish and humans, this animal model offers a unique opportunity to predict health risks associated with e-cigarette use.

The study by Yoon et al. is on the embryo-ototoxicity of a deodorant known as deodorant2 (DA2) [b]. In recent years, the results of several studies have demonstrated the toxicity of some types of deodorant due to the presence of substances such as triclosan or volatile organic compounds in their formulation, with them presenting an elevated risk for various organs such as the liver, skeletal muscle, and endocrine system. For this reason, the authors wanted to investigate the toxicity of DA2 on hair cells in zebrafish larvae through the exposition of this substance during the first days of embryological development, providing interesting insights into the ototoxic properties of this deodorant with serious implications for auditory health. The experimental design included a control group, a solvent control group to evaluate solvent toxicity, and an experimental group treated with DA2. The results showed that at 120 h post-fertilization, larvae exposed to deodorant at a concentration of 460 μg/mL demonstrated a significant reduction in hair cell count when compared to both the solvent control group and the control groups, with the results correlated with behavioral changes. The authors concluded that although zebrafish models do not fully replicate the complexity of the human system, the contribution of their study is significant in also drawing attention to the potential risks associated with the environmental diffusion of DA2 or similar compounds.

Bianchi et al. present a study focused on the synthesis and degradation of poly(ADP-ribose) in zebrafish brains after exposure to aluminum [c]. This study follows precedent research in which the toxicity of aluminum was demonstrated in embryos and zebrafish adults. In adult fish exposed to 11 mg/L Al, a concentration revealed in polluted sites, alterations of swimming ability and oxidative stress with PARP hyperactivation were revealed in the zebrafish brain, justifiable as an immediate response to oxidative DNA damage. In light of this evidence, for the first time in the brain of zebrafish exposed to this metal, Bianchi et al. studied the complete characterization of poly(ADPribosyl)ation system, including both the poly(ADPR) synthesis, catalyzed by PARP enzymes, and its degradation by PARGs in the brain of zebrafish adults. The results showed different PARP isoforms, among which the human counterpart PARP1 was also expressed. The highest PARP and PARG activity levels were measured after 10 and 15 days of exposure to metal, and the authors stated that PARP activation is related to DNA damage induced by Al whereas PARG activation is required to avoid PAR accumulation. On the contrary, PARP activity decreasing at longer exposure times indicates that neuronal cells could adopt the artifice of reducing polymer synthesis to avoid energy expenditure and allow cell survival. 

Another interesting paper included in the present Special Issue focus on mapping enzymes in zebrafish is the study of Yedji et al. In their study, the authors used zebrafish larvae, a model frequently used in ecotoxicology, to map, for the first time, serine hydrolase (SH) classes to reveal their involvement in the metabolism of xenobiotics such as dibutyl phthalate (DBP) [d]. The global activity of SHs was monitored in 5-day post-fertilization (dpf) zebrafish larvae exposed to different DBP concentrations to mimic low (5 µg/L, C5) and high (100 µg/L, C100) environmental contamination and the SH activity profile was compared with the control groups by also monitoring their survival and developmental abnormalities. The authors demonstrated the utility of the activity-based protein profiling (ABPP) approach to map active SHs in zebrafish larvae. The functional annotation of 49 SHs is common to all three conditions (control, C5, and C100), providing new information on the involvement of these enzymes in various metabolic pathways, identifying the carboxypeptidase CTSA as a potential biomarker for DBP exposure and additionally remarking on the necessity of further research on zebrafish to discover sensitive biomarkers. 

Always zebrafish larvae were used for the study of Hwang et al. [e] on the effects of polystyrene nanoparticles (PS-NPs). This is a study that concerns a popular research topic where the use of zebrafish as organism model is particularly diffuse. The widespread interest in micro- and nanoplastics is most notable in aquatic ecosystems, representing a significant problem for which it is important to ascertain the effective toxicity of all organisms. In this study, the authors exposed zebrafish larvae to two types of fluorescent polystyrene nanoparticles (PS-NPs) and showed that PS-NPs of 50 nm, unlike 100 nm PS-NPs, circulated in the blood vessels and accumulated in the brain, inducing abnormal behavioral patterns and changes in electroencephalograms. In addition, the quantification of endogenous neurochemicals in zebrafish larvae showed that 50 nm PS-NPs disturb dopaminergic metabolites. The study results were significant, with the authors concluding that the smallest nanoparticles disturb the nervous system.

The sixth paper included in the present Special Issue focuses on the heart of zebrafish adults. Karpushev et al.’s study is a physiological study aimed at analyzing the effects of empagliflozin, an inhibitor of sodium–glucose co-transporter 2 (iSGLT2) and drug used to improve cardiovascular outcomes in patients with and without diabetes that possesses antiarrhythmic activity [f]. The authors wanted to investigate the mechanisms, few elucidated, at the base of these protective effects by exploring the impact of this drug on the ventricular myocardium through analysis of ion channel activity and electrophysiological characteristics. In this study, the main cardiac ionic currents (I_Na_, I_CaL_, I_CaT,_ I_Kr_, and I_Ks_) and action potentials (APs) were analyzed. The authors have marked the utility of the zebrafish model to define the experimental study showing that empagliflozin has no effects on I_Na_, I_CaL_, and I_CaT_ in zebrafish ventricular cardiomyocytes but increases I_Kr_ and I_Ks_ currents and shortens AP duration in ventricular myocardium concluding that the cardioprotective action may be attributed to the upregulation effect on the I_K_ outward current.

In conclusion, the following Special Issue comprises a collection of interesting original research articles and one review paper that contribute to highlighting the effectiveness of the zebrafish model in investigating the action of pollutants or drugs. In addition, all these papers, relative to both zebrafish embryos and adult specimens, expand our understanding regarding relevant research topics, of great actuality and interest for the wider scientific community to incentive future studies using this animal model.
